# Do Insects Have Emotions? Some Insights from Bumble Bees

**DOI:** 10.3389/fnbeh.2017.00157

**Published:** 2017-08-23

**Authors:** David Baracchi, Mathieu Lihoreau, Martin Giurfa

**Affiliations:** ^1^Research Center on Animal Cognition, Center for Integrative Biology, Centre National de la Recherche Scientifique, University of Toulouse Toulouse, France; ^2^Laboratoire d'Ethologie Expérimentale et Comparée, Université Paris 13 Paris, France

**Keywords:** arousal, bumblebee, emotion-like state, insect cognition, internal states, invertebrates, motivation, reward

## Abstract

While our conceptual understanding of emotions is largely based on human subjective experiences, research in comparative cognition has shown growing interest in the existence and identification of “emotion-like” states in non-human animals. There is still ongoing debate about the nature of emotions in animals (especially invertebrates), and certainly their existence and the existence of certain expressive behaviors displaying internal emotional states raise a number of exciting and challenging questions. Interestingly, at least superficially, insects (bees and flies) seem to fulfill the basic requirements of emotional behavior. Yet, recent works go a step further by adopting terminologies and interpretational frameworks that could have been considered as crude anthropocentrism and that now seem acceptable in the scientific literature on invertebrate behavior and cognition. This change in paradigm requires, therefore, that the question of emotions in invertebrates is reconsidered from a cautious perspective and with parsimonious explanations. Here we review and discuss this controversial topic based on the recent finding that bumblebees experience positive emotions while experiencing unexpected sucrose rewards, but also incorporating a broader survey of recent literature in which similar claims have been done for other invertebrates. We maintain that caution is warranted before attributing emotion-like states to honey bees and bumble bees as some experimental caveats may undermine definitive conclusions. We suggest that interpreting many of these findings in terms of motivational drives may be less anthropocentrically biased and more cautious, at least until more careful experiments warrant the use of an emotion-related terminology.

Whether animals experience “emotions” is a controversial question that has long fascinated philosophers and scientists (Descartes and Rodis-Lewis, 1994; Darwin, 1998). The key to answering this question correctly is to define properly the term “emotions” (Gaulin and Mcburney, 2001; Russell, 2003; Barrett, 2006; Kron et al., 2015). In modern cognitive sciences, emotions are broadly defined as transient subjective states or processes that function in the management of goals and that involve three distinct components: a physiological response, a behavioral or expressive response and a subjective experience (Dantzer, 1990; Oatley and Johnson-Laird, 2014). They are described as intense, short-lived, reactions to specific events or stimuli that can be characterized by two main dimensional approaches (Russell, 1978, 2003; Kron et al., 2015): arousal (bodily activation or excitation) and valence (positive or pleasure and negative or displeasure).

While our conceptual understanding of emotions is largely based on human subjective experiences, research in comparative cognition has shown growing interest in the existence and identification of “emotion-like” states in non-human animals. Direct assessment of the subjective experience of emotions (i.e., the feeling) or the actual awareness of such states (i.e., the metacognitive aspect of emotions) is, at present, not possible in non-speaking subjects. Yet, there have been several attempts to use behavioral and physiological parameters as proxy indicators of animal emotions or more generally internal states (Paul et al., 2005). Accordingly, many behavioral tasks involving attention, perception, memory, expectation, and decision making, which are known to be influenced by emotional states in humans (Mathews and MacLeod, 1994; Lerner and Keltner, 2000), have been suggested to be reliable tools for assessing emotions across a wider range of animal species. In these tasks, scalability (gradation in intensity), persistence following stimulus or event cessation, valence (positive or negative), generalization to different contexts and stimulus degeneracy (different events or stimuli inducing the same behavior) have been used as main characteristics of emotionally driven behaviors (Anderson and Adolphs, 2014). Based on these operational definitions, the past 10 years have seen a burst of studies claiming the existence of emotions (or “emotion primitives” *sensu*, Anderson and Adolphs, 2014) in vertebrates such as fish (Rey et al., 2015), birds (Bateson and Matheson, 2007; Matheson et al., 2008; Valance et al., 2008), rats (Harding et al., 2004), pigs (Douglas et al., 2012), sheep (Doyle et al., 2010), goats (Baciadonna et al., 2016), and dogs (Mendl et al., 2010), among others (Baciadonna and McElligott, 2015).

## The case of invertebrates

This approach has been recently extended to invertebrates (Bateson et al., 2011; Fossat et al., 2014; Gibson et al., 2015; d'Ettorre et al., 2017). For example, it has been suggested that honey bees shaken in a confining tube, which supposedly simulates a predatory attack, show subsequent “pessimistic” cognitive biases in decision-making (Bateson et al., 2011; Schlüns et al., 2016). Crayfish subjected to electric shocks avoid aversive illuminated arms of an aquatic plus-maze, a behavior that was interpreted as an “anxiety-like state” (Fossat et al., 2014). Also fruit flies exposed to repetitive moving stimuli behave as if they were “frightened” and their internal state was described as being analogous to fear in humans (Gibson et al., 2015). These accounts overcome traditional views positing that invertebrates are simple automatons with reduced behavioral plasticity (Floreano et al., 2010). Yet, they go a step further by adopting terminologies and interpretational frameworks that could have been considered as crude anthropocentrism and that now seem acceptable in the scientific literature on invertebrate behavior and cognition. This change in paradigm requires, therefore, that the question of emotions in invertebrates is reconsidered from a cautious perspective and with parsimonious explanations.

## Emotional bumble bees?

In a recent issue of *Science*, Perry et al. (2016) have revived this discussion by claiming that bumble bees (*Bombus terrestris*) show a positive emotion–like state when they receive an unexpected sucrose reward, something that previous accounts on bee foraging behavior may simply have described as an increase in appetitive motivation (Núñez and Giurfa, 1996; Pankiw and Page, 2000; Sadler and Nieh, 2011). Perry et al. (2016) used the classical judgment bias paradigm (Harding et al., 2004), in which animals are trained to associate a stimulus A with a reward (CS+) and a stimulus B with the absence of reward, or a weak punishment (CS−). Thereafter, animals are tested in the presence of ambiguous stimuli that are intermediate between A and B (Harding et al., 2004). A positive emotion-like state is inferred if the animals tend to respond to the intermediate stimulus as if it were rewarding, i.e., closer to A, whereas a negative emotion-like state is inferred if the animals reject the intermediate stimulus, treating it as being equivalent to Perry et al. (2016) trained bumble bees to forage on two types of artificial flowers (blue or green) that were either rewarding (30% sucrose solution) or unrewarding (water only) and that were positioned at the opposite side of a test flight arena. After the training, they tested the bees with three additional ambiguous flowers, which were placed at intermediate positions and displayed colors lying between blue and green. One group of bees received an unexpected drop of concentrated (60%) sucrose solution upon leaving the nest just before entering the test arena, while control bees received nothing. In this way—the authors claim—bees experienced a positive emotion-like state induced by the sucrose consumption before the test.

Bees fed with an unexpected sucrose reward took less time to land on flowers with ambiguous colors, which was interpreted as an “optimistic” judgment of ambiguity. Importantly, the authors considered the possibility that sucrose may have simply excited the rewarded bees, thus resulting in higher exploration or faster choice of novel alternatives. To address this crucial point they measured both the speed of flight and the thorax temperature of tested bees. They discarded this possibility, although rewarded bees had a significantly higher thoracic temperature reflecting an increased foraging motivation (Stabentheiner and Hagmüller, 1991; Farina and Wainselboim, 2005; Sadler and Nieh, 2011) and the arena in which bees had to forage was probably too small (61 cm length, for an average flight speed of 4–5 m/s) to detect subtle differences in flight speed between control and rewarded bees. Given the wide range of flight speeds bees display in the field (Osborne et al., 2013), further experiments in a larger setup would be crucial to confirm these observations.

The unanticipated reward also induced bumble bees to reinitiate foraging faster after a simulated predator attack achieved via a mechanic trapping mechanism. The authors concluded that positive judgment biases could be generalized to different contexts, a fact which was said to support the definition of emotions in the case of bumble bees (Perry et al., 2016).

Finally, repeating the experiments after blockade of different biogenic-amine receptors suggested that dopamine was involved in the signaling of the unexpected sucrose reward. Indeed bees treated with an antagonist of the dopaminergic system were slower in their decisions despite having experienced the sucrose reward. It was concluded that dopamine mediates positive emotion-like state in bees. This conclusion is puzzling and remains to be confirmed as it contradicts current knowledge on reward signaling in bees. Indeed, although sucrose rewards are represented via a subset of dopaminergic neurons in the fruit fly brain (Burke et al., 2012; Liu et al., 2012), this has never been corroborated in bees where evidence indicates that octopaminergic neurons accomplish this function (Hammer, 1993; Hammer and Menzel, 1995, 1998; Farooqui et al., 2003). Interestingly, the authors aimed at blocking—without effects—the octopaminergic system but the blocker chosen to this end (mianserin) targets mainly the serotonergic system (Peroutka and Snyder, 1981) and is, therefore, not specific for octopamine receptors. This finding should be, therefore, verified using more specific blockers.

Previous research has established that insects, specifically bees, possess high levels of cognitive sophistication (Avarguès-Weber et al., 2011; Collett et al., 2013; Giurfa, 2013; Klein et al., 2017). From this perspective, it could be tempting to label these novel findings as emotions in bees, particularly because they seem to satisfy a number of the criteria identified by the operational definition of emotions, such as valence and generalization (Rolls, 2005; Anderson and Adolphs, 2014; Mendl and Paul, 2016). Yet, we maintain that despite this concordance, caution is warranted before attributing emotion-like states to honey bees and bumble bees as some experimental caveats (see above) may undermine definitive conclusions. Thus, a thorough reconsideration of the evidence available in the bumble bee work by Perry et al. (2016) let us conclude that from the components defining emotions, neither the subjective (hardly accessible) nor the physiological component (due to experimental incongruences, see above) have been definitively shown in bees. This underlines the necessity of a terminology deprived of subjective connotations when describing these results.

## Reinterpreting bumble bee performance in terms of appetitive motivation

Could a simpler explanation/terminology, like changes in motivation and arousal, suffice to describe the behavioral response of bees? While deconstructing the concept of emotions into basic building blocks (Anderson and Adolphs, 2014) partially circumvents the claim that the term emotions should be restricted to those states that include subjective feelings, interpreting many of these findings in terms of “motivational drives” may be less anthropomorphically biased and more cautious, at least until more careful experiments warrant the use of an emotion-related terminology. Indeed, the findings on bumble bees could be described as an increase in appetitive motivation due to an unexpected sucrose reward, which would lead the bees to accept more ambiguous alternatives than in the absence of such reward. Numerous works showing that bees increase their appetitive motivation and exhibit sensitized behavior upon unexpected positive rewards support this interpretation and terminology use (Núñez and Giurfa, 1996; Pankiw and Page, 2000; Sadler and Nieh, 2011). At this stage, no emotionally loaded terminology would thus be required to account for the reported performances of bees.

## Conclusion

Novel findings on bees, flies, and crayfish underline the interest of invertebrates for research addressing questions about internal states referring to canonical emotion primitives. Unravelling certain components of these primitives in invertebrates is possible given the tractability of their nervous circuits and brain structures (Menzel and Giurfa, 2001; Menzel, 2012). Future lines of research may attempt to characterize the brain activity (neuromodulation and/or activation of population neurons) of invertebrate animals facing behavioral tasks aimed at disentangling motivational from emotional explanations. In doing so, it should not be forgotten that neurotransmission and neuromodulation are not synonyms for emotions. Although there may be no emotional experience without participation of specific combinations of neuromodulators, emotional experiences cannot be simply equated to neuromodulator concentrations. This raises the difficulty of employing terminologies for which not only subjective components are hardly accessible but also for which neural circuitries do not allow homologies and straightforward parallels with those of vertebrates, in particular of humans. In the light of these facts, caution is needed before adopting terminologies which may hamper the objective analysis of animal behavior.

## Author contributions

DB has conceived the study. All authors listed have made a substantial and intellectual contribution to the work, have written the manuscript and approved it for publication.

### Conflict of interest statement

The authors declare that the research was conducted in the absence of any commercial or financial relationships that could be construed as a potential conflict of interest.
